# Continental Notes from a Holiday Correspondent

**Published:** 1894-07-21

**Authors:** 


### CONTINENTAL NOTES FROM A HOLIDAY CORRESPONDENT.

HEIDELBERG.

No University town in Germany can offer greater attractions to the medical visitor than does Heidelberg, for in addition to its endless historical and artistic attractions there is now a most flourishing medical school. The fact that within the last year or so one Heidelberg professor has refused a call to Berlin and two have refused calls to Vienna indicates the feeling of the Germans themselves towards this city. The University was founded more than 500 years ago, but for many centuries was entirely under clerical control, and the medical faculty never flourished. At the beginning of this century, however, after having been ruined by the French, it was restored and completely reorganised by the Grand Duke Charles Frederick, Elector of Baden. He introduced the broad and tolerant principle that in every vacancy in any faculty the best candidate should be appointed, regardless of religious qualifications. From that time to this the University has steadily grown and prospered, and the medical faculty has shared in the general prosperity. It is now said to be one of the two most successful and best manned faculties at the University. The medical buildings lie in a group in the new part of the town, between the railway station and the river. They are most complete, comprising general hospital, and children's, women's, and opthalmic hospitals. All these are fitted up in modern style, with every facility for teaching and laboratory work, &c. The patients, though of course not so many as in large capitals, are still more numerous than one would expect in such a small town, as they come from all the surrounding country.

In general surgery the chief attraction is Professor Czerny, whose name has been so much before the profession lately, as the probable successor to Billroth. I heard that his refusal to leave Heidelberg was due partly to his great love for the quaint old town, and not only to difficulties about sufficient laboratories, &c., in Vienna, and this I can well believe, for the devotion of the inhabitants to their town is most marked, though hardly to be wondered at.

Tke Frauenklinik is presided over by Geheimrath Professor Kehrer, who speaks English fairly well, and makes strangers very welcome. There are two resident assistants. The building is capitally fitted up for the special work done there; the theatres are specially good. The strict antisepsis usually seen in Germany is carefully carried out in all operative procedures.

The Augenklinik attracts more strangers than any other department of the medical work I believe. In one room, for instance, I saw an Irishman, an American, and a Dane working together.

Professor Leber, the chief of the Augenklinik, is an elderly man, but still very vigorous. He speaks a little English, and is most courteous to strangers. I had the pleasure of accompanying him round his wards on several occasions, but there did not happen to be any case of extraordinary interest in at the time. It was rather amusing to see the military style of his inspection. On his coming into each ward we discovered all the patients who were up—perhaps twelve or fifteen—drawn up in line, the tallest at one end, and the shortest at the other, in a descending series. The only point I specially noticed in the wards was the arrangement of Leiter's tubes in each, supplied from pipes on the walls, so that they could be applied to any patient in the ward in a few moments.

Professor Leber's fame rests chiefly on his splendid pathological work, so that this department is of special interest. It is elaborately fitted up with several laboratories; one for microscopical work, another for bacteriology, another for experimental work, and so on. In the museum are about 2,200 specimens, sections of most of which may be cut by those working in the laboratory, provided they write out a full account of their investigation of each one for the use of Professor Leber. Unfortunately it is not very easy to get into the laboratory as a worker. One must first take a practical course with one of the assistants for five or six weeks, and then, if one works well, one may be allowed to work in the laboratory. In addition to those studying medicine a number of English and Americans are always found working at the University, and especially at the splendid chemical laboratories in the Wrede Platz, as the fame of Professor Victor Meyer attracts men from all lands. The medical or scientific visitor to Heidelberg should not miss hearing him lecture, which he does at eight o'clock every morning during the summer session; his class-room, filled with students of every nationality, is a sight worth seeing.

